# Special Issue “Functional and Structural Genomics Studies for Plant Breeding”

**DOI:** 10.3390/ijms27125206

**Published:** 2026-06-09

**Authors:** Alberto Vangelisti, Tommaso Giordani, Andrea Cavallini

**Affiliations:** Department of Agriculture, Food and Environment (DAFE), University of Pisa, Via del Borghetto, 80, 56124 Pisa, Italy; alberto.vangelisti@unipi.it (A.V.); tommaso.giordani@unipi.it (T.G.)

## 1. Introduction

The rapid development of high-throughput sequencing technologies and the integration of omics sciences have markedly advanced plant genomics over the past decade. Structural and functional genomic analyses now enable an increasingly precise dissection of the genetic basis of complex agronomic traits. These approaches are becoming, and in many cases have already become, central to plant breeding, particularly in the context of climate change, with the associated emergence of biotic and abiotic stresses, and the need to sustainably enhance crop productivity and quality.

Indeed, the integration of structural and functional genomics is progressively transforming breeding strategies, shifting from predominantly descriptive studies toward predictive frameworks. In this regard, several practical applications are emerging.

One of the first major applications is genomic selection, which leverages increasingly dense genome-wide marker datasets and improved statistical models to predict the genetic value of alleles underlying traits with even highly complex genetic architectures, with greater accuracy [1].

Equally important are pan-genome-based approaches, which enable the identification of structural variants, presence–absence variants, and copy number variations that escape detection in analyses relying on a single reference genome [2]. In fact, recent studies demonstrate that structural variants play a significant role in stress adaptation and disease resistance. In this context, pan-genomic information enhances the identification and utilization of alleles derived from wild relatives and landraces for introgression into breeding programs.

The transition from the discovery of loci underlying traits of interest to their effective improvement is further facilitated by the integration of QTL/GWAS mapping with precision breeding based on CRISPR technologies [3]. The latest CRISPR toolkits, including base editing and prime editing [4], enable increasingly precise allele modifications and are being widely employed to improve yield components, tolerance to abiotic stresses, and quality-related traits in major crops.

Multi-omics integration has also made substantial progress in recent years, supported by advances in network inference strategies and machine learning-based integration methods [5]. These approaches allow the identification of regulatory hubs and gene–environment interactions, which are essential for the development of cultivars resilient to climate change.

Finally, the integration of artificial intelligence and high-throughput phenotyping with genomic data is reshaping predictive breeding [6]. Deep learning and hybrid statistical–machine learning models are increasingly being applied to integrate genotypic, phenotypic, and environmental data, enhancing predictive accuracy across diverse environments and accelerating selection processes.

## 2. Overview of the Special Issue Contributions

A key aspect of breeding innovation is the characterization and exploitation of genetic diversity. In the Special Issue, Cañas-Gutiérrez et al. (contribution 1) performed whole-genome resequencing of 205 avocado trees, providing detailed insights into genomic patterns underlying genetic divergence across the Americas. Their analyses elucidate population structure and identify genomic regions contributing to differentiation among avocado cultivars, offering valuable information for germplasm conservation and strategic parental selection.

The generation of novel variation is also important. In this respect, a paper in the Special Issue reports on a carbon-ion-beam-induced soybean mutant population, characterized through genome-wide analysis and revealing extensive sequence and structural variation (contribution 2). This work shows the potential of heavy-ion mutagenesis to expand allelic diversity beyond natural variation, thus enriching breeding pools with new functional alleles.

Quantitative trait dissection remains fundamental for crop improvement and was the object of two studies. Wang et al. (contribution 3) mapped QTLs for fiber- and seed-related traits using chromosome segment substitution lines derived from *Gossypium hirsutum* × *Gossypium darwinii*. Their results highlight the value of introgression from wild species and of high-resolution mapping populations for identifying loci controlling agronomic performance. In another study, Suo et al. (contribution 4) functionally characterized the R2R3 MYB transcription factor GhMYB201, demonstrating its role in promoting cotton fiber elongation through regulation of cell wall loosening and very-long-chain fatty acid biosynthesis.

Finally, two studies analyzed gene regulatory networks, beyond single-gene analyses. Ma et al. (contribution 5) deciphered the gene regulatory network controlling flower development in spinach, identifying major transcription factors and regulatory modules involved in reproductive development. Similarly, Simoni et al. (contribution 6) identified coordinated regulatory networks controlling phenolic compounds and steviol glycoside biosynthesis in *Stevia rebaudiana*, providing insight into metabolic pathway integration relevant to quality traits.

## 3. Conclusions

In practical breeding programs, genomic approaches are implemented sequentially: (i) characterization of genetic diversity through structural genomics analyses; (ii) identification of loci and inference of regulatory networks underlying target traits; (iii) functional validation of genetic variants through gene editing; and (iv) predictive genomic selection. The integration of these components represents a realistic and operational roadmap for next-generation plant breeding programs. The studies included in this Special Issue collectively illustrate components of this pipeline, demonstrating how structural and functional genomics can combine to accelerate crop improvement, exemplifying how genome-wide approaches can be applied to characterize genetic diversity, identify loci controlling agronomic traits, and elucidate regulatory mechanisms relevant to crop improvement.
